# Temperature-responsive regulation of the fermentation of hypocrellin A by *Shiraia bambusicola* (GDMCC 60438)

**DOI:** 10.1186/s12934-022-01862-w

**Published:** 2022-07-05

**Authors:** Yongdi Wen, Baosheng Liao, Xiaoxiao Yan, Zhenqiang Wu, Xiaofei Tian

**Affiliations:** 1 Guangdong Key Laboratory of Fermentation & Enzyme Engineering, School of Biology and Biological Engineering, South China University of Technology, 382 East Out Loop, University Park, Guangzhou, 510006 China; 2 The Second Clinical College, Guangzhou University of Chinese Medicine, 232 East Out Loop, University Park, Guangzhou, 510006 China; 3 Zhuhai Institute of Modern Industrial Innovation, South China University of Technology, 8 Fushan Road, Fushan Industrial Park, Zhuhai, 519100 China

**Keywords:** Hypocrellin A biosynthesis, Temperature, RNA-seq, Expression, Mycelial morphology

## Abstract

**Background:**

Hypocrellin A (HA) is a perylene quinone pigment with high medicinal value that is produced by *Shiraia bambusicola* Henn. (*S. bambusicola*) and *Hypocrella bambusae* (Berk. & Broome) Sacc. (Ascomycetes) with great potential in clinical photodynamic therapy. Submerged cultivation of *S. bambusicola* is a popular technique for HA production. However, there is not much research on how temperature changes lead to differential yields of HA production.

**Results:**

The temperature regulation of submerged fermentation is an efficient approach to promote HA productivity. After a 32 °C fermentation, the HA content in the mycelia *S. bambusicola* (GDMCC 60438) was increased by more than three- and fivefold when compared to that at 28 °C and 26 °C, respectively. RNA sequencing (RNA-seq) analysis showed that the regulation of the expression of transcription factors and genes essential for HA biosynthesis could be induced by high temperature. Among the 496 differentially expressed genes (DEGs) explicitly expressed at 32 °C, the hub genes *MH01c06g0046321* and *MH01c11g0073001* in the coexpression network may affect HA biosynthesis and cytoarchitecture, respectively. Moreover, five genes, i.e., *MH01c01g0006641*, *MH01c03g0017691*, *MH01c04g0029531*, *MH01c04g0030701* and *MH01c22g0111101*, potentially related to HA synthesis also exhibited significantly higher expression levels. Morphological observation showed that the autolysis inside the mycelial pellets tightly composted intertwined mycelia without apparent holes.

**Conclusions:**

The obtained results provide an effective strategy in the submerged fermentation of *S. bambusicola* for improved HA production and reveal an alternative regulatory network responsive to the biosynthesis metabolism of HA in response to environmental signals.

**Supplementary Information:**

The online version contains supplementary material available at 10.1186/s12934-022-01862-w.

## Background

*Shiraia bambusicola* Henn and *Hypocrella bambusae* (Berk. & Broome.) Sacc. are ascomycetes, and both mainly parasitize branches of bamboos such as *Brachystachyum densiflorum* (Rendle) Keng and *Fargesia spathacea* Franch [1, 2]. With high medicinal value, their stroma is rich in hypocrellins (HYP) and polysaccharides and 11,11′-dideoxyverticilin [3–6]. HYP is a natural photosensitive perylenequinone that generally includes derivatives of hypocrellin A (HA), hypocrellin B (HB), hypocrellin C (HC), and hypocrellin D (HD). The HA and HB compounds have shown pharmacological functions in photodynamic therapy (PDT) with visible-light-induced antitumor, antiviral, and antibacterial activities [7–10]. However, natural HA products are insufficient to meet the growing research and market demand due to restrictions of natural stromal resources. Although the HYP biological synthesis pathways were unclear, submerged fermentation with *S. bambusicola* has excellent application prospects in producing HYP [11]. Several strategies for improving the production of HYP have been developed through gene editing or breeding the strains, light or chemical stimulation, and optimization of medium carbon/nitrogen sources [12–14]. For example, overexpressing the *O-methyltransferase*/*FAD-dependent monooxygenase* gene and *hydroxylase* gene increased the production of HA by 200% and 100%, respectively. Through a 5-min ultrasonic stimulation at 40 kHz, HA content could be increased by 177.2%, while adding 0.6% (v/v) Triton X-100 induced HYP production to reach 780.6 mg/L in submerged culture. In nature, environmental temperature is a crucial factor influencing mycelium growth and the synthesis of fungal secondary metabolites through a complex regulatory metabolic network [15, 16]. Anderson et al. revealed a remarkable morphological change in the conidia and mycelia formed by *Aspergillus niger* by a temperature increase [17]. Furthermore, Zhou et al. found that controlling temperature was beneficial to mycelium growth during the fermentation of *Monascus* sp. [18]. In addition, heat shock caused the synthesis of two new natural compounds, aspernidine A and B, by *Aspergillus nidulans* [19]. It is believed that the physiological or metabolite biosynthesis responses to temperature regulation can be contributed by activating or inhibiting the expression of the functional gene clusters in fungi [20, 21]. However, there is not much research on how temperature changes lead to differential yields of HA production during the submerged cultivation of *S. bambusicola.* The *multicopper oxidase* gene, *fasciclin* gene, *polyketide synthase* gene, *o-methyltransferase*/*FAD-dependent monooxygenase* gene, *O-methyltransferase* gene, *hydroxylase* gene and *FAD/FMN-dependent oxidoreductase* gene were closely related to the biosynthesis of the HYPs according to the comparative transcriptomic analysis of wild-type strains and UV-irradiated mutants [22]. RNA sequencing (RNA-seq) was a popular technique for determining the expression of biosynthesis genes for HYPs. Through the RNA-seq method, it was revealed that Triton X-100 improved HA yields by inducing the gene expression of *polyketide synthase*, *cytochrome P450* and *demethylsterigmatocystin 6-O-methyltransferase* during the submerged cultivation of *S. bambusicola* [11].

To investigate the effects of temperature regulation on the yield of HA biosynthesis, we performed a comparison of the HA yields, mycelial morphology and mycelial pellet structure in submerged cultivation of *S. bambusicola* (GDMCC 60438) at 26 °C, 28 °C and 32 °C, respectively. In addition, a possible mechanism for promoting HA production under higher temperatures was proposed through further identification and functional annotations of differentially expressed genes (DEGs) by the RNA-seq method. It was helpful in further elucidating the regulation mechanism of the biosynthetic pathway of HA and providing feasible strategies for effectively improving the yield of HA.

## Materials and methods

### Submerged fermentation of HA by *S. bambusicola* (GDMCC 60438)

The *S. bambusicola* (GDMCC 60438) strain was cultivated at 28 °C for 10 days on a potato dextrose agar (PDA) plate (Huankai Microbial Sci & Tech Co., Ltd, Guangzhou, China). After washing with water containing 0.1% (v/v) Tween 80 (Damao Chemical Reagent Factory, Tianjin, China), the mycelium suspensions were transferred to a 250-mL conical flask (Shubo Group Co., Ltd, Chengdu, China) containing 50 mL potato dextrose broth (PDB) media (Huankai Microbial Sci & Tech Co., Ltd, Guangzhou, China). The seed culture was cultivated at 28 °C for 60 h at 150 rpm in a rotary incubator (Zhicheng Analytical Instrument Manufacturing Co., Ltd, Shanghai, China). Following inoculation with a 10% (v/v) seed suspension in a 250 mL baffled-bottom flask (Shubo Group Co., Ltd, Chengdu, China) containing 50 mL of fermentation medium with 4 g/L potato extract (Yuanye Bio-Technology Co., Ltd, Shanghai, China), 12 g/L beef extract (Huankai Microbial Sci & Tech Co., Ltd, Guangzhou, China) and 10 g/L glycerol (Macklin Biochemical Co., Ltd, Shanghai, China), submerged fermentation was performed at 26, 28, and 32 °C for 3 days at 150 rpm.

### Determination of biomass and HA content

The mycelial samples were collected from the broth by SHZ-D (III) vacuum filtration (Ketai Instrument Equipment Co., Ltd, Guangzhou, China). After washing with 30 mL of distilled water 3 times, the mycelia were freeze-dried using a SCIENTZ-10 N freeze dryer (Scientz Biotechnology Co., Ltd, Ningbo, China) for 48 h until constant weight. The mass of the mycelia was measured using a Shimadzu UX620H balance (Shimadzu Corporation, Shimane-ken, Japan). The HA content in the mycelia was determined by the high-performance liquid chromatography (HPLC) method [23]. A total of 0.05 g of dried mycelia was transferred to 10 mL tubes containing 5 mL of dichloromethane (Zhiyuan Reagent Co., Ltd, Tianjin, China) and thoroughly ground into powder with silicon sand (60 mesh, Guangzhou Chemical Reagent Factory, Guangdong, China). The HA was extracted from the mixture under ultrasound mixing (40 kHz, 600 W) in a 100S ultrasonic cleaner machine (Chaojie Technology Industrial Co., Ltd, Shenzhen, China) for 30 min. Before HPLC analysis, the HA solution was filtered through 0.22 µm nylon 66 filters (ø13 mm, Jinteng Experimental Equipment Co., Ltd, Tianjin, China). HPLC analysis was performed using an e2695 system (Waters Corporation, Milford, MA, USA) equipped with an ODS column (5 µm × 4.6 mm × 250 mm, Shimadzu Corporation, Shimane-ken, Japan) and a 2998 PDA detector (Waters Corporation, Milford, MA, USA). A mixture of methanol (Aladdin Bio-Chem Technology Co., Ltd, Shanghai, China) and acetic acid aqueous solution (pH = 2) (9:1, v/v) was used as the mobile phase with a flow rate of 1 mL/min. The column temperature was 30 ℃, and the injection volume was 10 µL. The wavelength of the detector was set at 467 nm. The HA content in mycelia and HA yield were calculated by Eqs. 1 and 2 as follows:

1 $$\text{HA content in mycelia (mg/g) =\,}\frac{\text{HA concentration (mg/mL)} \times {\text{solvent volume (mL)}}}{\text {ground mycelia (g)}}$$

2 $$\text{HA yield (mg/L) =\,}\frac{\text{HA\,content\,in\,mycelia\,(mg/g)} \times {\text{total\,mycelia\,biomass\,(g)}}}{\text{fermentation\,broth\,volume\,(L)}}$$

### Morphological observation of the paraffin section (PS)

The PS was determined/prepared based on a modified method [24, 25]. Fresh mycelial pellets (0.5 g) were fixed in 5 mL of FAA fixative containing 50% (v/v) aqueous ethanol solution, glacial acetic acid (Zhiyuan Reagent Co., Ltd, Tianjin, China) and then formaldehyde solution (Chemical Reagent Factory, Guangzhou, China) (18:1:1, v/v/v) for 24 h. After fixation, the pellets were prestained with 5 mL of lactophenol methyl blue reagent (Leagene Biotech Co., Ltd, Beijing, China) for 12 h. Then, the samples were dehydrated using 25, 50, 70, 80, 96 and 100% aqueous ethanol solution successively for 20 min, embedded in paraffin blocks, sectioned, and mounted on glass slides. After restaining with 1% (v/v) crystal violet dyeing solution (Bkman Biotechnology Co., Ltd, Changde, China) for 5 min, the internal morphology and structure of the mycelial pellets were observed in the bright field using an Olympus IX83 inverted microscope (Olympus Corporation, Tokyo, Japan) with 100 IOS and an exposure time of 37.04 μs at magnifications of 40× and 100×.

### Scanning electron microscopy (SEM) observation

The mycelial pellets were collected from the fermentation broth at 8000 rpm for 5 min in a 5804 R centrifuge (Eppendorf Corporation, Hamburg, Germany). After successively washing with distilled water and 0.1 M phosphate buffer saline solution (PBS) for 3 times, the pellets were dispersed in 5 mL of 2.5% glutaraldehyde (Phygene Bio-Technology Co., Ltd, Fuzhou, China) for 4 h and rinsed with 0.1 M PBS to remove residual glutaraldehyde. Then, the mycelial pellets were suspended in 0.1 M PBS and frozen at – 80 °C for 1 h. Finally, the mycelium was freeze-dried using a 10 N freeze dryer (Scientz Biotechnology Co., Ltd, Ningbo, China) and coated with conducting film by a 150TES sputter coater (Electron Microscopy Sciences Corporation, Hatfield, UK). The surface morphology of the mycelium was observed by a Merlin compact field emission SEM (Carl Zeiss Medical Technology Inc., Oberkochen, Germany) with a scanning voltage of 5.00 kV [26].

### RNA extraction and RNA sequencing library construction

Total RNA was extracted using a MiniBEST Plant RNA Extraction Kit (Takara Biotechnology Inc., Kusatsu, Japan). Approximately 100 mg of fresh mycelia was ground in liquid nitrogen before extraction. The RNA quality and quantity were determined using the Thermo Scientific NanoDrop 2000 Spectrophotometer (Thermo Fisher Scientific, Waltham, MA, USA).

RNA-seq of the *S. bambusicola* (GDMCC 60438) mycelia at three different fermentation temperatures was performed by Annoroad Gene Technology Co., Ltd. (Beijing, China), and three replicates for each sample were used for RNA extraction. A cDNA library was constructed for each replicate, and the libraries were sequenced on an MGI T7 platform (BGI Inc., Shenzhen, China) followed by paired-end 150-bp read generation. Then, the low-quality reads were filtered out using FastQC (http://www.bioinformatics.babraham.ac.uk/projects/fastqc/) and Skewer (https://sourceforge.net/projects/skewer) with the parameters set to -q 20 -Q 30 -l 50 [27].

### RNA sequencing data analysis

Gene transcript levels (TPM values) at different temperatures were calculated by HISAT2 (v2.2.1) and StringTie (v2.2.0) (http://ccb.jhu.edu/software.shtml) [28]. Then, the coding sequences of genes were annotated functionally in the Pfam database (http://pfam.xfam.org/). The DEGs among the samples at different temperatures were identified by the R package DESeq2 (v3.8) [29]. Cluster analysis was performed using the pheatmap package (https://cran.r-project.org/web/packages/pheatmap/) according to DEGs’ TPM values [30]. Furthermore, the DEGs were assigned to the Gene Ontology (GO) categories using the database (http://www.geneontology.org), while significantly enriched GO terms by DEGs were determined coupled with *KS* < 0.01 using clusterProfiler (v4.0) [31]. In addition, the coexpression network of DEGs was constructed by an R package, weighted-correlation-network analysis (v1.70-3) [32].

### Quantitative reverse-transcription PCR (qRT–PCR) validation of RNA-Seq data

qRT–PCR was used to verify the high expression of DEGs at 32 °C revealed by RNA-seq analysis. cDNA was synthesized with RNA as a template using the PrimeScriptTM RT reagent Kit with gDNA Eraser (Takara Biotechnology Inc., Kusatsu, Japan). Gene-specific forward/reverse primers were designed using Primer Premier 5 (Additional file 1). The 20 μL qRT–PCR system contained 0.8 μL of each primer, 2 μL of cDNA and 10 μL of mix of TB Green^®^ Premix Ex Taq™ II (Tli RNaseH Plus) (Takara Biotechnology Inc., Kusatsu, Japan) and 6.4 μL of RNase-free water. The qRT–PCR amplification procedure was set to 95 °C for 30 s followed by 40 cycles of 95 °C for 5 s and 60 °C for 20 s on Light Cycler 96 fluorescence quantitative PCR equipment (F. Hoffmann-La Roche Ltd, Basel, Switzerland). The 2-∆∆CT method was used to calculate the relative expression level of DEGs. *MH01c13g0084281* was used as the internal reference gene. Three biological and three technical replicates were analyzed for each reaction.

### Statistical analysis

The significance of differences among means regarding the HA content and mycelium biomass from 3 temperature treatment levels was analyzed with one-way ANOVA followed by Tamhane’s T2/Dunnett’s T3/Games-Howell and LSD tests using IBM SPSS Statistics 26. The significance level was *p* < 0.05.

## Results and discussion

### Effects of temperature on HA yield and mycelial growth

**Fig. 1:**
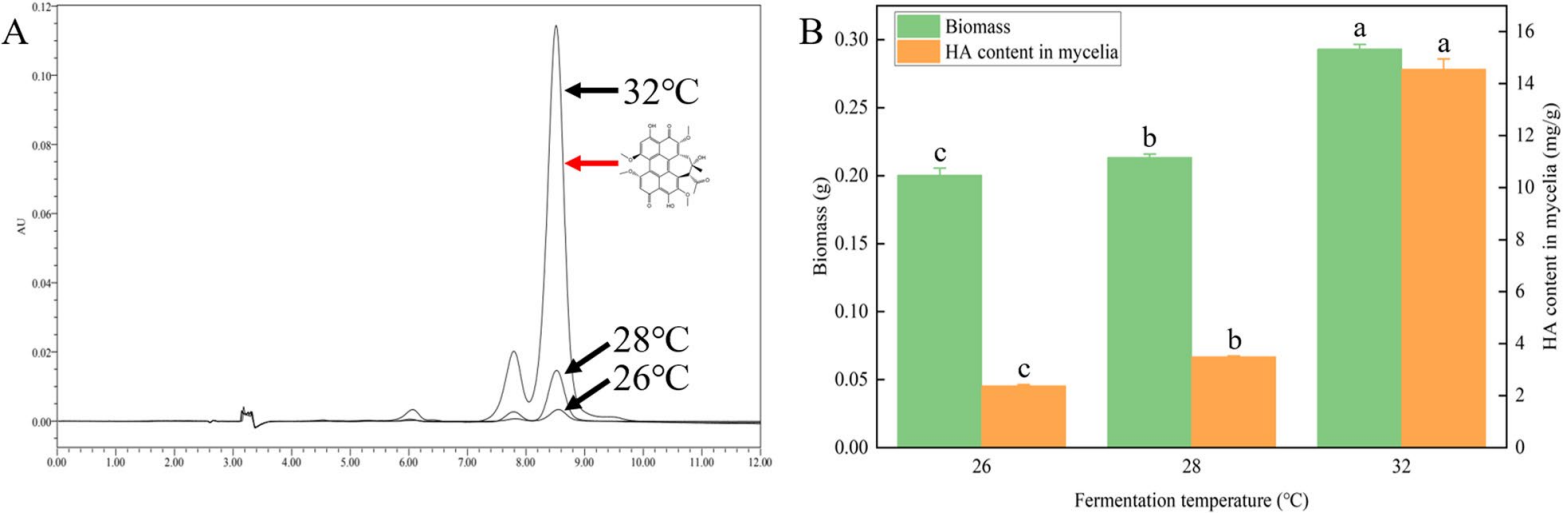
Effects of different temperatures on *S. bambusicola* (GDMCC 60438) growth and HA content. **A** HPLC analysis of HYP at 26, 28 and 32 °C. Black arrow, chromatograms at different temperatures; red arrow, the chromatographic peak of HA. **B** Biomass and HA content in mycelia at 26, 28 and 32 °C. The letters above the bars indicate significant differences (*p* < 0.05)

As shown in Fig. 1A, the enhancement of temperature has a considerable influence on improving the HA production of *S. bambusicola* (GDMCC 60438). The mycelial biomass at 28 °C was slightly larger than that at 26 °C, while the mycelial biomass at 32 °C was clearly higher than those at 26 and 28 °C (Fig. 1B). Additionally, compared to those at 26 °C or 28 °C, the HA content in mycelia at 32 °C was significantly promoted by 600% and 400%, respectively (*p* < 0.05). Temperature is an essential factor in submerged fermentation and affects fungal growth, morphology, secondary metabolite production, and the formation of new substances [33–35]. Optimized temperature can be distinct but truly depends on the biosynthesis pathways and transcription factors conducive to the production of secondary metabolites. For instance, reduced temperature can enhance aflatoxin production in *A. parasiticus,* while higher temperature benefits the biosynthesis of sterigmatocystin by *A. nidulans* [36]. This study demonstrated that high temperature has a positive effect on both HA content and mycelial growth during submerged cultivation. In the place of *S. bambusicola* origin, stroma formation concomitant with HYP accumulation only occurs under an average air temperature above 25 °C [37]. These results are also consistent with the thermophilic nature of *S. bambusicola* in switching energy and carbon flux toward the production of secondary metabolites. Although multiple methods, such as overexpression of *O-methyltransferase*/*FAD-dependent monooxygenase*, introducing exogenous sodium nitroprusside and ultrasonic stimulation, were developed to improve HA production by 156–200% [12, 13, 38], appropriately enhancing the temperature is a simple and effective strategy for significantly improving HA synthesis through submerged cultivation.

### Changes in the mycelial morphology and internal morphology of mycelial pellets

**Fig. 2:**
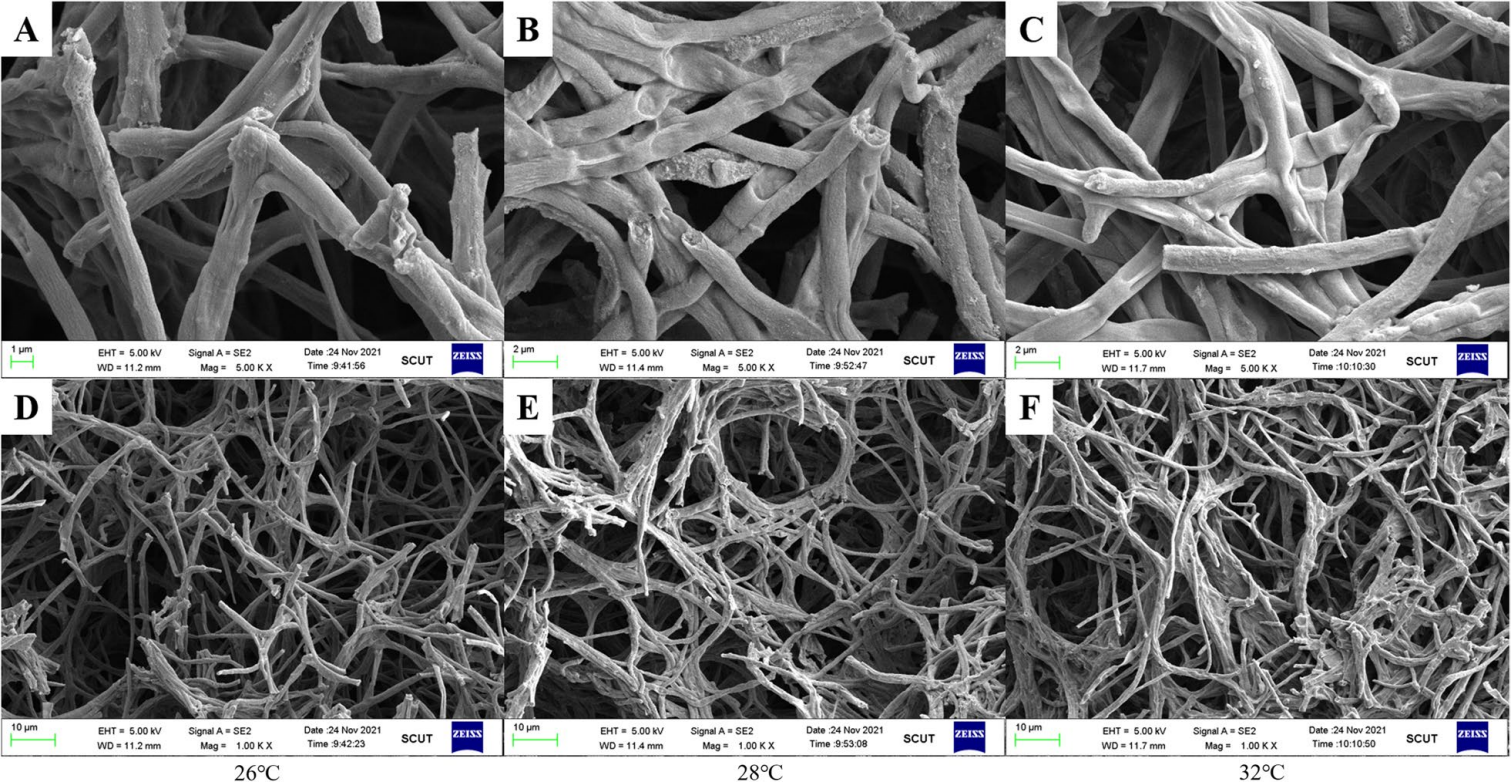
Observation of the surface morphology of mycelial pellets at different temperatures by SEM. Mycelial morphology **A** At 26 °C, bar = 1 μm. **B** At 28 °C, bar = 2 μm. **C** At 32 °C, bar = 2 μm. **D** At 26 °C, bar = 10 μm. **E** At 28 °C, bar = 10 μm. **F** At 32 °C, bar = 10 μm

The mycelial morphology and winding on the surface of mycelial pellets were observed by SEM. At different fermentation temperatures, with increasing temperature, the delicate veins on the mycelial surface decreased, the surface became smoother, and the hyphae adhered to each other more closely (Fig. 2A–C). It was shown that a small portion of the hyphae loosely gathered with more gaps between them at 26 ℃ (Fig. 2A and D). When the temperature was increased to 28 °C, multiple hyphae were parallelly twisted to form a more uniform network structure with smaller gaps between the hyphae (Fig. 2B and E). When the temperature was further increased to 32 °C, multiple mycelia adhesions were found to bind together and intertwine to form a denser surface structure with fewer gaps (Fig. 2C and F). Combined with observations of mycelial morphology, our results showed that temperature is one of the critical factors affecting mycelial morphology during submerged cultivation.

**Fig. 3:**
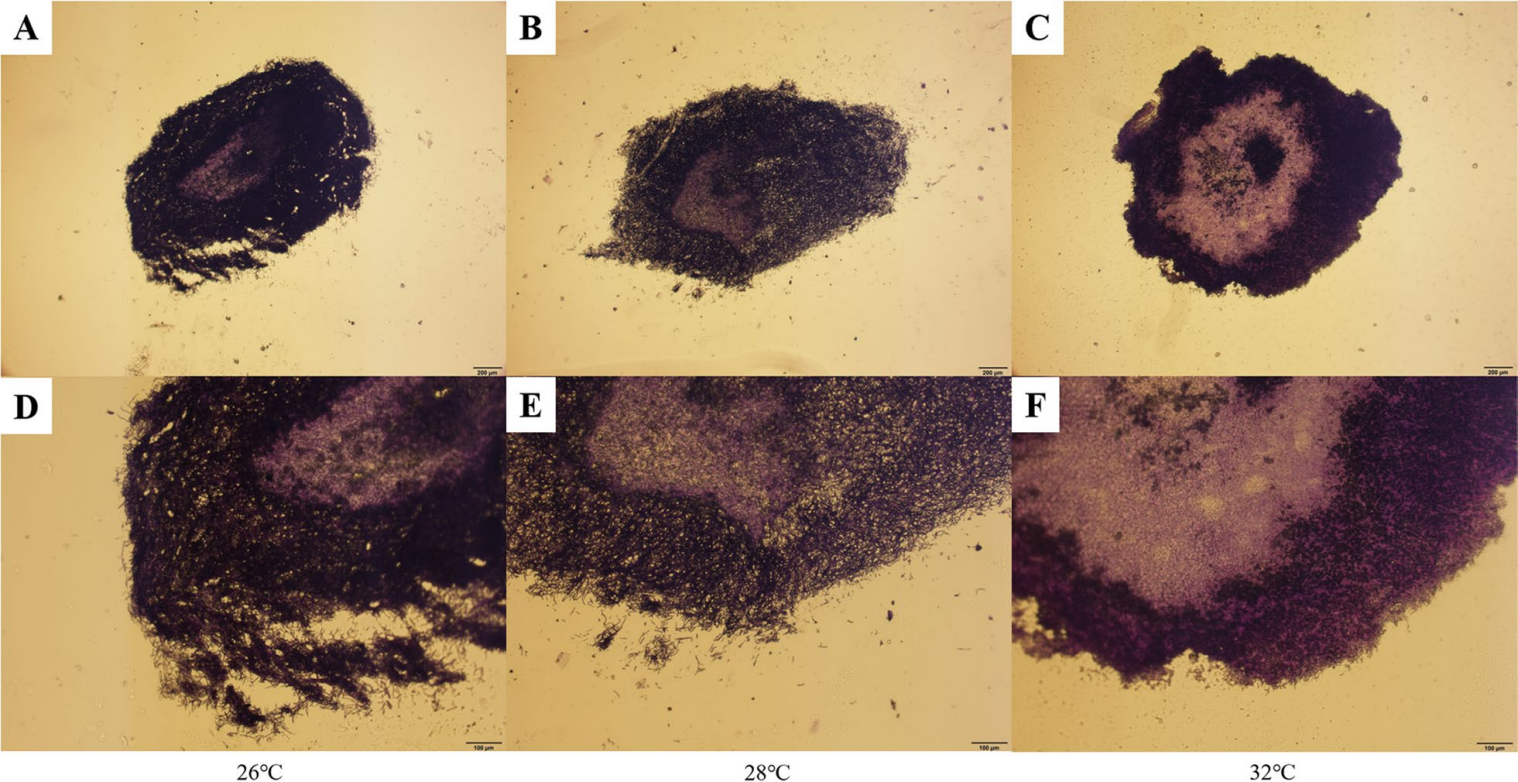
Observation of the internal morphology of mycelial pellets at different temperatures with PS. **A** Morphology of mycelial pellet at 26 °C, bar = 200 μm. **B** Morphology of mycelial pellet at 28 °C, bar = 200 μm. **C** Morphology of mycelial pellet at 32 °C, bar = 200 μm. **D** Morphology of mycelial pellet at 26 °C, bar = 100 μm. **E** Morphology of mycelial pellet at 28 °C, bar = 100 μm. **F** Morphology of mycelial pellet at 32 °C, bar = 100 μm

Although the mycelia of filamentous fungi produced secondary metabolites in morphological forms of clump, dispersed mycelium and pellet in submerged fermentation [39], the mycelial pellet was the general morphological form through the submerged fermentation of HA by the *Shiraia* fungi. The morphology of pellets is beneficial to the production of antibiotics, organic acids, and enzyme preparations by fungi [40–42]. It was revealed that the structure of the mycelial pellets could affect the accumulation and secretion of secondary metabolites [43]. For instance, higher polygalacturonidase could be achieved from denser *A. niger* pellets [44]. Through the microscopic observation of the cross-section of mycelial pellets, the active growing and nongrowing regions of pellets were distinguished (Fig. 3). The mycelia close to the external layer and central regions were clearly separated by a thin layer of transition both at 26 °C and 28 °C, whereas this transition region was not evident in the mycelial pellet at 32 °C (Fig. 3A–C). Contrasting the peripheral layer of the mycelial pellets, there were many large voids and cavities between the hyphae at 26 °C, while the hyphae became loosely twisted with reduced gaps at 28 °C, and more interestingly, mycelia were tightly intertwined at 32 °C. Fragments of the mycelia appeared, demonstrating the occurrence of autolysis in the central area of the pellet [45]. Only limited autolysis was observed in the central structure of the pellets at both 26 °C and 28 °C. However, the pellets became a hollow structure by the intensive autolysis of the hyphae at 32 °C (Fig. 3D–F).

**Fig. 4:**
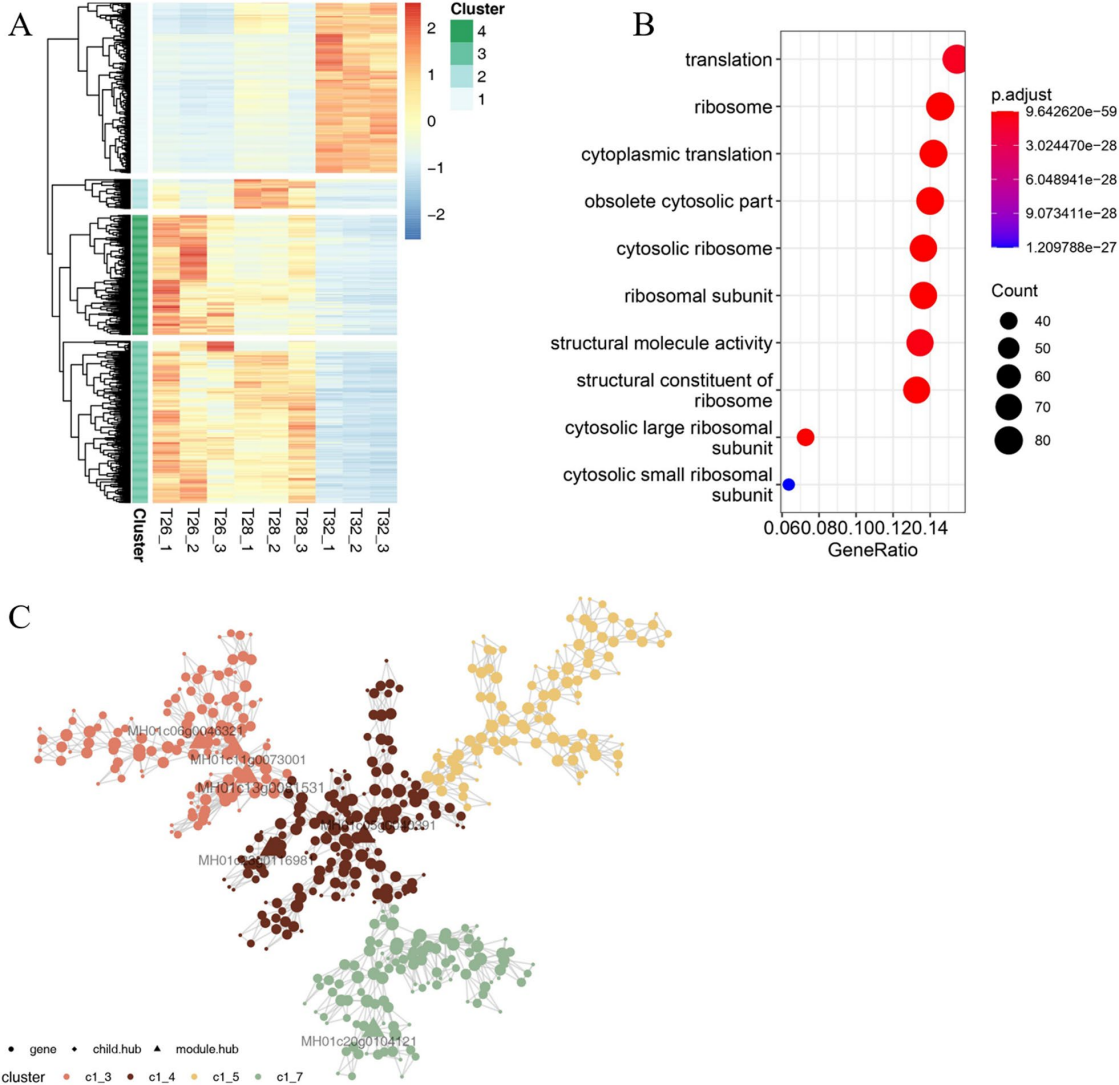
Analysis of DEG expression patterns and functions. **A** DEG expression levels at 26, 28 and 32 °C. **B** GO functional enrichment of DEGs. **C** Coexpression network of DEGs

This study revealed that the formation of mycelial pellets by tightly intertwined mycelia may be conducive to HA synthesis (Fig. 2). Mycelia in the outer layer of mycelial pellets could be of high activity due to relatively sufficient nutrients and oxygen supply. In contrast, dense mycelia are intertwined to limit the transfer of nutrients and oxygen to the interior of the mycelial pellets, resulting in the autolysis of the mycelia in the central part [46, 47]. In our study, the observation of PS exhibited an intensive autolysis of inner mycelia in the pellets at 32 °C compared to that at 26 °C or 28 °C (Fig. 3). It is believed that temperature can regulate the structure of the mycelial pellets, probably by affecting morphology-related gene expression. For instance, the *MH01c11g0073001* hub gene was identified through coexpression analysis of the highly expressed DEGs at 32 °C. It is believed that proteins encoded by *MH01c11g0073001* contain the LIM domain (Fig. 4C and Table 1), which regulates cytoarchitecture, cell adhesion, signal transduction and gene expression [48]. Moreover, it was recognized that the pellet porosity is inversely proportional to its density [49]. Accelerated growth of the mycelia under higher temperatures would cause a tightly twisted mycelial structure and deterioration of the lack of nutrients and oxygen within the pellet. As a result, autolysis occurs more rapidly to generate hydrolyzed molecules as additional nutrients to the growth of the pellet. The hydrolyzed hyphae may also generate molecules for signal transduction for HA synthesis [50–52].

**Table 1:** Pfam functional annotation of hub genes in the coexpression network

Gene ID	Pfam ID	Description
MH01c06g0046321	PF00172.17	Zn(II)Cys_6_-type zinc finger protein
	PF04082.17	Fungal specific transcription factor
MH01c11g0073001	PF00412.21	LIM domain protein
	PF00620.26	GTPase activating protein
MH01c13g0081531	PF00320.26	GATA zinc finger protein
	PF08550.9	Unknown function domain
MH01c23g0116981	PF00324.20	Amino acid permease
MH01c05g0040391	PF00069.24	Protein kinase
MH01c20g0104121	–	–

–: No Pfam annotation

### Differential expression pattern enrichment and coexpression analysis of the DEGs between different temperatures

A total of 12,350 genes were identified (Additional file 2), of which 7348 genes (59.50%) were identified from the Pfam database, and 4216 genes contained multiple Pfam annotations (Additional file 3). To identify the differences in gene expression in a more intuitive manner, a hierarchical clustering analysis was conducted. The expression patterns of a total of 1326 DEGs between different temperatures can be grouped into 4 clusters. Of note, 469 DEGs highly expressed at 32 °C were specifically distinguished in Cluster 1 against other DEGs with higher expression at 28 °C or 26 °C (Fig. 4A, Additional file 4 and Additional file 5). Furthermore, GO enrichment analysis of the specifically highly expressed DEGs at 32 °C was performed. However, these identified DEGs had no significant GO enrichment, while the GO terms of 261 DEGs from Clusters 2, 3, and 4 were primarily enriched in the functions of translation, ribosome, cytoplasmic translation, obsolete cytosolic part, cytosolic ribosome, ribosomal subunit, structural molecule activity, and structural constituent of ribosome (Fig. 4B, Additional file 6). The coexpression analysis showed that most of the specifically highly expressed DEGs at 32 °C could be clustered into a module that consisted of 4 submodules. The 5 hub genes that link multiple other genes in the module were annotated as Zn(II)Cys_6_ type zinc finger protein/fungal specific transcription factor *(MH01c06g0046321*), LIM domain protein/GTPase activating protein (*MH01c11g0073001*), GATA zinc finger protein (*MH01c13g0081531*), amino acid permease (*MH01c23g0116981*), and protein kinase (*MH01c05g0040391*) (Table 1). However, there were no hub genes found in the submodules (Fig. 4C and Additional file 7).

### Gene expression involved in HA biosynthesis and modification

**Fig. 5:**
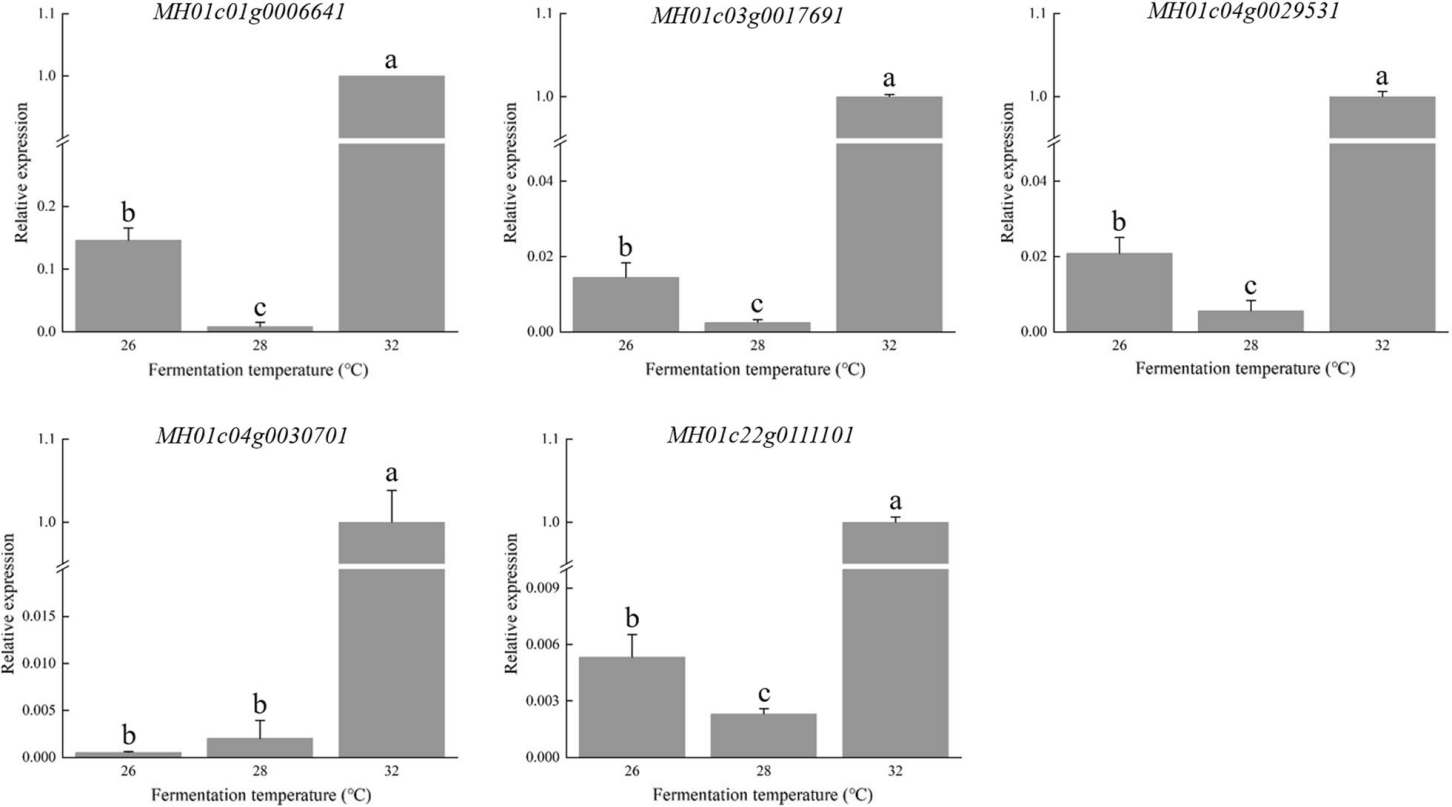
Relative expression levels of several DEGs at different temperatures by qRT–PCR determination. The different letters above the bar indicate significant differences (*p* < 0.05)

The putative pathway for HA was proposed by Zhao et al. [22]. Starting from the substrate acetyl-coenzyme A and malonyl-coenzyme A, HA can be produced through a series of catalytic reactions such as polymerization, cyclization, and oxidation [22, 53]. Genes encoding polyketide synthase (PKS), O-/FAD-dependent methyltransferase, monooxygenase, and FAD/FMN-dependent oxidoreductase may be directly involved in HA biosynthesis [54, 55]. The RNA-seq data demonstrated that FAD-dependent oxidoreductase (*MH01c01g0006641*), O-methyltransferase (*MH01c03g0017691*), FAD-binding monooxygenase (*MH01c04g0029531*), cytochrome P450 (*MH01c04g0030701*) and polyketide synthase (*MH01c22g0111101*) were significantly upregulated when the temperature was increased from 26 to 32 °C. They may have directly functioned in HA biosynthesis (Fig. 5, Table 2).

**Table 2:** Pfam functional annotation of HA biosynthetic genes in qRT–PCR determination

GeneID	Pfam ID	Description
MH01c01g0006641	PF01565.22	FAD-dependent oxidoreductase
MH01c03g0017691	PF00891.17	O-methyltransferase
MH01c04g0029531	PF01494.18	FAD-dependent monooxygenase
MH01c04g0030701	PF00067.21	Cytochrome P450
MH01c22g0111101	PF08659.9	Polyketide synthase

Moreover, the zinc finger transcription factor, which could also regulate the expression of HA biosynthesis genes, was also found to be upregulated. It was believed that the expression levels of genes involved in the HA synthesis pathway were upregulated by a Zn(II)Cys_6_ activating transcription factor that was overexpressed in *S. bambusicola* S4201 [56]. Among the hub genes in our coexpression network, the highly expressed gene *MH01c06g0046321* was annotated as a Zn(II)Cys_6_-type zinc finger protein (Fig. 4C and Table 1). Its expression can be promoted by the temperature increase to 32 °C, contributing to improved HA accumulation in the mycelia by upregulating the pathway gene expression for HA biosynthesis.

To further evaluate the validity of the obtained results, these 5 DEGs were selected for expression level examination by qRT–PCR. Compared with the expression at 26 °C and 28 °C, all abovementioned representative genes exhibited significantly higher expression at 32 °C (Fig. 5). The overall trend of relative expression levels was consistent with that of the RNA sequencing. It was determined that genes involved in pathways (such as polyketide biosynthesis, methyl transduction, oxygen transduction and oxidoreduction) were directly correlated with HA accumulation in the mycelia.

## Conclusions

This study performed RNA-seq on *S. bambusicola* (GDMCC 60438) at different fermentation temperatures. Furthermore, it was found that the significant increase in HA production caused by higher temperature may be due to the influence of higher temperature on the HA synthesis pathway and mycelial morphology through analyzing expression levels, building a coexpression network and scanning function annotation of identified DEGs as well as observing the microscopic morphology of mycelial pellets. Exploring the mechanism of higher temperature to increase HA yield is of great value to mine the essential genes affecting HA synthesis and analyze the biosynthesis pathway of HA.

## Supplementary Information

**Additional file 1: Table S1.** Forward and reverse primers used in qRT–PCR.

**Additional file 2: Table S2.** Identified genes of *S. bambusicola* (GDMCC 60438) and their TPM values.

**Additional file 3: Table S3.** Pfam annotation of genes of *S. bambusicola* (GDMCC 60438).

**Additional file 4: Table S4.** DEGs of Cluster 1 in the heatmap and their TPM values.

**Additional file 5: Table S5.** DEGs of Clusters 2, 3 and 4 in the heatmap and their TPM values.

**Additional file 6: Table S6.** GO enrichment of DEGs of Clusters 2, 3 and 4.

**Additional file 7: Table S7.** DEGs in coexpression network analysis.

## Data Availability

All data generated or analyzed during this study are included in this published article [and its Additional files].

## Abbreviations

DEGs: Differentially expressed genes
GO: Gene ontology
HA: Hypocrellin A
HB: Hypocrellin B
HC: Hypocrellin C
HD: Hypocrellin D
HPLC: High-performance liquid chromatography
HYP: Hypocrellins
PBS: Phosphate buffer saline
PDA: Potato dextrose agar
PDB: Potato dextrose broth
PDT: Photodynamic therapy
PKS: Polyketide synthase
PS: Paraffin section
qRT–PCR: Quantitative reverse-transcription PCR
RNA-seq: RNA sequencing
*S. bambusicola*: *Shiraia bambusicola*
SEM: Scanning electron microscopy
